# Chronic Enteropathy in Dogs—Epidemiologic Aspects and Clinical Characteristics of Dogs Presenting at Two Swedish Animal Hospitals

**DOI:** 10.3390/ani12121507

**Published:** 2022-06-09

**Authors:** Johanna Holmberg, Lena Pelander, Ingrid Ljungvall, Caroline Harlos, Thomas Spillmann, Jens Häggström

**Affiliations:** 1 Department of Clinical Sciences, Swedish University of Agricultural Sciences, Almas allé 8, 750 07 Uppsala, Sweden; lena.pelander@slu.se (L.P.); ingrid.ljungvall@slu.se (I.L.); jens.haggstrom@slu.se (J.H.); 2 Anicura Albano Animal Hospital, Rinkebyvägen 21B, 182 36 Danderyd, Sweden; caroline.harlos@anicura.se; 3 Departments of Faculty of Veterinary Medicine, Equine and Small Animal Medicine, University of Helsinki, PL 57 Koetilantie 2, 00014 Helsinki, Finland; thomas.spillmann@helsinki.fi

**Keywords:** chronic enteropathy, canine, prevalence, clinical study, Sweden

## Abstract

**Simple Summary:**

Canine chronic enteropathy is characterized by persistent (>3 weeks) or recurring gastrointestinal signs, such as diarrhea, vomiting, loss of appetite, and weight loss. Depending on treatment response, chronic enteropathy is classified as food-responsive-, antibiotic-responsive-, immunosuppressant-responsive-, or non-responsive enteropathy. Information about prevalence and breed disposition of dogs with chronic enteropathy is limited. The aim of this retrospective study was to investigate period prevalence, breed distribution, and characterization of chronic enteropathy in dogs presenting at two Swedish animal hospitals. A total of 814 dogs met inclusion criteria and the period prevalence of chronic enteropathy was 1.1% of the total number of dogs. Breeds with the highest relative risk included Norwegian Lundehund, West Highland White Terrier, and Miniature Poodle. Treatment outcome was classified in 72.9% of dogs, and characterized as immunosuppressant-responsive (55.2%), food-responsive (11.4%), non-responsive (5.2%), and antibiotic-responsive (1.1%). Non-responsive dogs were more likely to present with anemia, hypoproteinemia, hypoalbuminemia, increased C-reactive protein concentrations, and ascites.

**Abstract:**

Information about prevalence and breed predisposition of canine chronic enteropathy (CE) is limited. The aim of this retrospective study was to investigate period prevalence, breed disposition, clinical features, diagnostic results, and treatment response of CE in dogs presenting at two Swedish animal hospitals during 2013–2018. A medical record search was performed to identify CE dogs including those with ≥3 visits because of gastrointestinal disease and/or that had undergone gastroduodenoscopy/colonoscopy during 2013–2018. Dog characteristics, case history, physical examination, laboratory variables, therapeutic protocol, and treatment response were recorded. Inclusion criteria for CE were met by 814 dogs. Period prevalence of CE was 1.1% of total number of dogs. Breeds with the highest relative risk included Norwegian Lundehund, West Highland White Terrier, and Miniature Poodle. Median age at presentation was 3.8 (IQR 1.8–6.8) years. French Bulldogs and Miniature Schnauzers presented at a younger age (<2.5 years) compared to other breeds (*p <* 0.05). In a subset of dogs, serum hypoalbuminemia (116/662, 17.5%), hypocobalaminemia (98/647, 15.1%), and increased C-reactive protein (CRP) concentrations (145/267, 54.3%) were diagnosed. Treatment outcome was classified in 72.9% of dogs and characterized as immunosuppressant-responsive (55.2%), food-responsive (11.4%), non-responsive (5.2%), and antibiotic-responsive (1.1%). Non-responsive dogs were more likely to present with anemia hypoproteinemia/albuminemia, increased CRP, and ascites (*p <* 0.05). In conclusion, the prevalence of dogs with CE at Swedish hospitals agreed with earlier reports, but risk breeds differed slightly and, compared to other breeds, a younger age of CE onset was found in two breeds. The largest proportion of dogs was immunosuppressant-responsive and the smallest antibiotic-responsive.

## 1. Introduction

Canine chronic enteropathy (CE) is characterized by persistent (>3 weeks) or recurring gastrointestinal signs, such as diarrhea, vomiting, loss of appetite, and weight loss [1,2,3]. The pathophysiology of CE is not completely known but is thought to include a complex interplay between host genetics, environmental factors, the intestinal microenvironment, and the immune system [4,5]. Several breeds have been reported to be predisposed to CE, supporting the role of host genetics [6,7,8,9,10,11]. The diagnosis of CE is made after having excluded other medical conditions causing chronic gastrointestinal signs, such as parasitic or infectious disease, endocrine, hepatic, pancreatic, renal disease, or mechanical obstruction [2,12,13]. CE is, depending on treatment response, classified as food-responsive (FRE), antibiotic-responsive (ARE), immunosuppressant-responsive (IRE), or non-responsive (NRE) [1,3,14,15]. If there is a protein loss across the intestinal wall leading to hypoalbuminemia, the condition is termed protein-losing enteropathy (PLE) [2,16,17]. Two different clinical scoring systems, called Canine Inflammatory Bowel Disease Activity Index (CIBDAI) and Canine Chronic Enteropathy Clinical Activity Index (CCECAI), have been developed to evaluate the clinical disease activity in dogs with CE [14,18]. Hypoalbuminemia, hypocobalaminemia, hypovitaminosis D, marked endoscopic lesions in the duodenum, and high CIBDAI are recognized as negative prognostic factors in canine CE [14,18,19,20].

The term inflammatory bowel disease (IBD) is also used in veterinary medicine for dogs where intestinal inflammation has been diagnosed by histological examination [2,21]. Advantages with the use of the term CE instead of IBD is that CE can be used for animals in which intestinal inflammation is suspected, but in which no histological examination have been performed, and the terminology does not infer which treatment that will be needed to control clinical signs [2]. Several treatment interventions have been suggested for dogs with CE, where the use of elimination diets, immunosuppressant treatment with glucocorticoid agents, as well as probiotic treatment, are supported by strong scientific evidence [15,22,23]. Budesonide, which is a nonhalogenated glucocorticoid with less systemic adverse effects, have shown to be as effective as prednisone or induction therapy of IBD in dogs [24].

Information about prevalence of CE in dogs is scarce both at the general practice and referral center level [1], and information about treatment response and breed disposition of dogs with increased risk of CE is limited [6]. Only a few studies have reported treatment outcomes >6 months [3,14,20,22,25,26,27]. The primary aim of this retrospective study was therefore to investigate the period prevalence of canine CE at two Swedish animal hospitals, as well as to investigate dog characteristics, breed disposition, diagnostic results, therapeutic protocol, and treatment response. A secondary aim was to compare laboratory variables between dogs in the different treatment response groups (FRE, IRE, ARE, and NRE).

## 2. Materials and Methods

### 2.1. Study Population

In this retrospective study, a medical record search was performed at two large Swedish animal hospitals: Anicura Albano Animal Hospital (AAAH) in Stockholm and the University Animal Hospital (UAH) at the Swedish University of Agricultural Sciences in Uppsala, to identify dogs with CE. Initially, the diagnostic index of the medical record system Trofast (Version 8.6.0.0, Trofast AB, Västerås, Sweden) was used to search the database for dogs that were presented at either animal hospital because of gastrointestinal signs at ≥3 occasions between January 2013 and December 2018. Dogs were also included if the first visit because of GI signs had taken place before January 2013. A search was performed in the database for diagnostic codes related to vomiting, diarrhea, abdominal pain, weight loss, anorexia, tenesmus, and gastrointestinal inflammation. In addition, a search was performed for dogs that had underwent gastroduodenoscopy/colonoscopy during the same time period. Case duplicates were removed from the resulting data set and the remaining medical records were scrutinized by a veterinarian (JHo). Dogs that had plausible clinical signs of CE, such as persistent (>3 weeks) or recurring diarrhea, vomiting, loss of appetite, or weight loss, were included in the study. Dogs were also included if they still had clinical signs of CE despite a successful treatment of a confirmed intestinal parasitic infection, with negative fecal examination results at the control examination. Gastrointestinal biopsies were not required for the dogs to be included. Dogs were excluded from the study if they had visited the animal hospitals because of acute gastrointestinal disease or if they had been diagnosed with primary endocrine, hepatic, pancreatic or renal disease, infectious or parasitic disease, mechanical obstruction, or a macroscopic neoplastic lesion.

### 2.2. Database

Dog characteristics (breed, sex, age at first and last visit, body weight (BW) and body condition score (BCS)), case history, physical examination, laboratory variables, and therapeutic protocol and response, were documented in the database. The date of the first visit because of CE was noted, as well as date and reason for the last visit at the animal hospital (including also non-CE related reasons), regardless of being performed during the study period or later. Information about activity level, appetite, stool consistency, and stool frequency were included, as well as information regarding presence of vomiting, abdominal pain, weight loss, or pruritus. Results of laboratory variables including fecal and blood samples, as well as findings from the abdominal ultrasound examination, gastroduodenoscopy/colonoscopy, and histological examination were also recorded. When blood sample results from the initial CE related visit were available in the medical record database, these results were documented together with the reference range used by the laboratory that performed the examinations. Treatment information regarding diet brand, probiotics, type of medication, and concurrent medical conditions was noted from the time of the first CE related visit until the last documented visit in the medical record. In addition, treatment response as well as time and reason for euthanasia/death was documented in the data spread sheet.

### 2.3. Classification of Dogs

The CE was classified as FRE, IRE, ARE, or NRE depending on treatment response as previously described (1, 2, 14). In short, the dogs were classified with FRE if they had an adequate response to diet change and needed no additional treatment, and classified with IRE if they did not respond solely to diet change but had an adequate response to immunosuppressant treatment. They were classified with ARE if they had an inadequate response to diet change and immunosuppressant treatment, and solely responded to antibiotic treatment. The dogs were classified with NRE if they did not respond to either diet change, immunosuppressant treatment, or antibiotic treatment. For the individual dog, the response was assessed at the discretion of the veterinarian scrutinizing the medical records (JHo), and only classified in the database if the information could clearly be obtained from the medical record (≥2 weeks follow up). If the information regarding treatment response was not available, or if the dog was treated with immunosuppressant and antibiotic treatment concurrently, the dog was categorized as non-classified.

### 2.4. Statistical Methods

Descriptive statistical calculations were performed in JMP Pro (v16.0, Cary NC). Proportions, medians, and interquartile ranges (IQR) are reported. Relative risk (RR) and 95% confidence intervals for developing CE for each breed was calculated by using the following formula: RR = (number of dogs of a given breed with CE/total number of dogs with CE)/(number of dogs of a given breed/total number of dogs). Differences in age of onset between different breeds and treatment classification groups were tested using Wilcoxon rank-sum test, because age of onset was found not to be normally distributed using the F-test. Numerical laboratory blood analysis data was dichotomized into either above (C-reactive protein [CRP], leukocytes, creatinine, urea, alanine aminotransferase), below (albumin, total protein, leukocytes, hematocrit, calcium, folic acid, cobalamin), or within reference interval. Comparisons between categorical data were performed using either the Chi-2 or the Fischer’s exact two-tailed tests. Subanalyses were performed with pairwise comparisons if the overall *p*-value was <0.05, and one or both variables included >2 groups using the Fischer’s exact test with Bonferroni correction for multiple comparisons.

## 3. Results

### 3.1. Study Population

**Figure 1:**
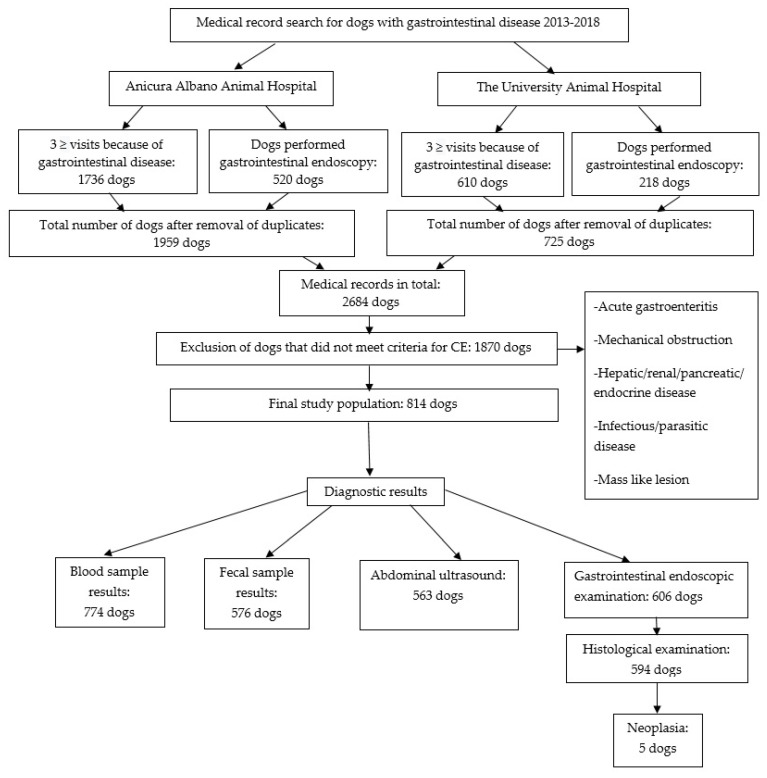
Flowchart describing the establishment of the final study population of 814 dogs and number of individual dogs that had undergone specific tests in this group.

A flowchart of the different steps in the search process of the medical records at AAAH and UAH is presented in Figure 1. The final study population comprised 814 dogs. In total, 16.2% (132/814) of the dogs were referred to AAAH and UAH from other clinics, and the rest of the dogs presented as primary cases. Thirty of the included dogs had an intestinal parasitic infection at the time of presentation. These dogs had continuous signs of CE despite a successful treatment with negative fecal examination results at the control examination, and they were therefore included in the data set. Five dogs were diagnosed with neoplasia at the histological examination but had no signs of macroscopic neoplastic lesions at the abdominal ultrasound or gastrointestinal endoscopic examination. These five dogs were included in the study population because histological examination was not required for inclusion. The final study population comprised 463 males and 351 females, of which 136 of the male dogs were neutered, and 114 of the female dogs were spayed. In total, 138 different breeds were represented in the data set, and the most common breeds were Mixed Breed (107), Golden Retriever (35), French Bulldog (34), Cavalier King Charles Spaniel (CKCS) (31), Rottweiler (30), Labrador Retriever (27), German Shepherd (25), Miniature Poodle (25), Chihuahua (25), and Miniature Schnauzer (21). Dogs presented at a median age of 3.8 (IQR 1.8–6.7) years, ranging from 0.2–13.8 years. French Bulldogs and Miniature Schnauzers presented at a younger age (below 2.5 years) compared to other breeds (*p* < 0.05). The median time of follow up from the first visit until the last visit was 2.8 (IQR 0.9–4.6) years. The longest time of follow up was 12.6 years. In total, 30.0% (244/814) of the dogs had been euthanized, and 0.8% (7/814) of the dogs were reported to have died at home. In 47.5% (116/244) of the dogs that were euthanized, CE was the reported reason for euthanasia. For the dogs that were euthanized or were reported to have died at home, the median time of follow up from the first CE related visit until time of death was 2.8 (IQR 0.9–4.6) years. General information about the study population and presenting signs are presented in Table 1.

### 3.2. Period Prevalence and Relative Risk

**Figure 2:**
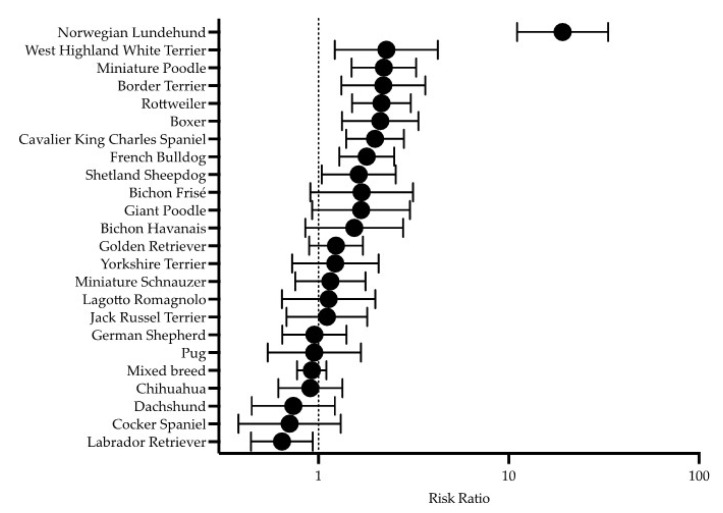
Forest plot showing the relative risk of chronic enteropathy (CE) and 95% confidence intervals breeds represented by ≥10 dogs in the dataset. See statistical methods for details and notice the logarithmically transformed *x*-axis.

During the study period, a total of 77,142 dogs visited the animal hospitals: 48,914 dogs at AAAH and 28,228 dogs at UAH. The total number of dogs that presented with gastrointestinal signs during the same period was 18,409 dogs: 12 024 dogs at AAAH and 6,385 dogs at the UAH. The period prevalence of CE was 1.1% (814/77 142) of the total number of dogs that visited the animal hospitals. The CE dogs represented 4.4% (814/18 409) of all dogs that presented at the animal hospitals with gastrointestinal signs during the study period. The breeds with an increased RR of presenting with CE were Norwegian Lundehund, West Highland White Terrier (WHWT), Miniature Poodle, Border Terrier, Rottweiler, Boxer, CKCS, French Bulldog, and Shetland Sheepdog (Figure 2).

### 3.3. Classification of Dogs

Treatment outcome could be classified in 72.9% (593/814) of the dogs, and their CE was characterized as IRE (449/814, 55.2%), FRE (93/814, 11.4%), NRE (42/814, 5.2%), and ARE (9/814, 1.1%). Twenty-seven percent (221/814) of the dogs were not assigned a classification because of lack of follow up after the treatment start, information about treatment response could not be clearly obtained from the medical record, or due to concurrent initial treatment with immunosuppressant and antibiotic agents. Among the non-responsive dogs, 41/42 (97.5%) of the dogs were euthanized due to CE, and 1/42 (2.4%) of the non-responsive dogs were euthanized due to pneumonia. Non-responsive dogs were more likely to be euthanized due to CE than dogs with FRE, IRE, and dogs that were not assigned a classification (*p* < 0.008). Norwegian Lundehunds were more likely to be non-responsive than food-responsive (*p* < 0.008). There was no significant difference in treatment outcome among the other breeds.

### 3.4. Diagnostic Results

**Figure 3:**
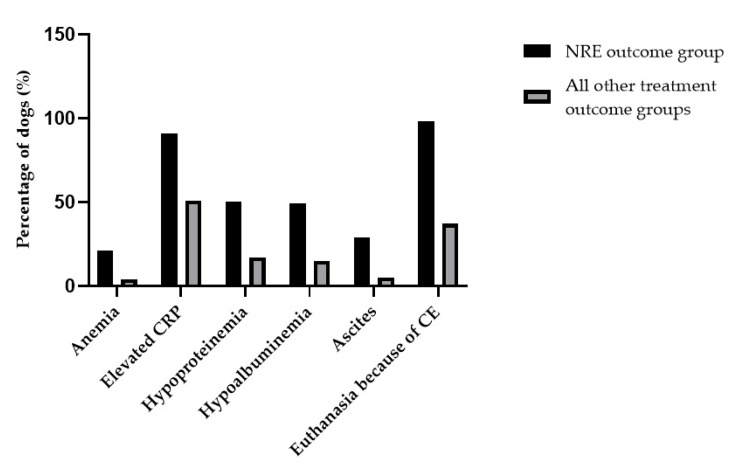
Bar graph that illustrates the characteristics of dogs in the NRE outcome group contrasted to the dogs belonging to the other treatment outcome groups (FRE, IRE, ARE, and non-classified outcome group).

Results of hematology and/or biochemistry were available in 95% (774/814) of the dogs (Table 2). Abnormal results such as anemia (32/632, 5.1%), hypoalbuminemia (116/662, 17.5%), hypoproteinemia (125/655, 19.1%), hypocobalaminemia (98/647, 15.1%), and increased CRP concentrations (145/267, 54.3%) were identified. Dogs with NRE were more likely to present with hypoproteinemia, hypoalbuminemia, and anemia compared to the FRE, IRE, and non-classified outcome groups (*p* < 0.008). There was no significant difference in total protein, albumin, or hematocrit concentrations among the FRE-, IRE- and non-classified outcome groups. Dogs with NRE were more likely to present with increased CRP concentrations compared to the IRE outcome group (*p* < 0.008), but no significant difference in CRP concentrations was seen among the NRE, FRE, and non-classified outcome groups. The ARE outcome group was excluded from the multiple comparison test because of an insufficient number of dogs. Results of abdominal ultrasounds were documented in 69.2% (563/814) of the dogs, and in 59.5% (335/563) of these dogs, there was an ultrasonographic abnormality recorded in the gastrointestinal tract. Information regarding presence of ascites was documented in 67.6% (550/814) of the dogs and was identified in 6.7% (37/550) of them (Table 2). Dogs with NRE were more likely to present with ascites compared to the FRE, IRE, and non-classified outcome groups (*p* < 0.008). A graph that illustrates the characteristics of dogs in the NRE outcome group contrasted to the dogs belonging to the other treatment outcome groups is presented in Figure 3. A gastroscopy and/or colonoscopy was performed in 74.4% (606/814) of the dogs and histological results were available from 98.0% (594/606) of the gastrointestinal endoscopic examinations. Gastrointestinal endoscopy and histological findings are presented in Table 3.

### 3.5. Treatment

A hydrolyzed diet was initially administered to 69.2% (563/814) of the dogs, whereas 13.0% (106/814) were fed a novel protein diet. The rest of the dogs were given either gastrointestinal diet (111/814, 13.6%), home-prepared food (18/814, 2.2%), or other types of diet that were chosen because of concurrent diseases or due to the dog owner’s preference (14/814, 1.7%). In 82.0% (668/814) of the dogs, immunosuppressive treatment consisted of prednisolone/methylprednisolone, and in 16.2% (132/814), the prednisolone/methylprednisolone was combined with additional immunosuppressive treatment (Table 4). In 63.0% (512/814) of the dogs, gastroprotectant treatment with either proton pump inhibitors, sucralfate, or famotidine were administered (Table 4).

Antibiotic treatment was used in 41.9% (341/814) of the dogs, and the most common antibiotic substances used were metronidazole (325/341, 95.3%) and amoxicillin (40/341, 11.7%). In 10.3% (35/341) of the dogs treated with antibiotics, other antibiotic agents were administered (tylosin, enrofloxacin, amoxicillin plus clavulanic acid, or doxycycline). Among the dogs that received antibiotics, 46.2% (158/341) were not assigned a classification due to concurrent initial treatment with antibiotic and immunosuppressive therapy. In 40.6% (138/341) of the dogs, there was an inadequate response to the antibiotic treatment but an adequate response to immunosuppressant treatment, whereby these dogs were classified in the IRE outcome group. Of the dogs that received antibiotic treatment, 8% (28/341) were classified with NRE, 2.6% (9/341) were classified with ARE, and 2.4% (8/341) were classified in the FRE outcome group. Of the dogs with NRE, 92.9% (39/42) were treated with prednisolone/methylprednisolone, and in 35.7% (15/42) of the dogs, the treatment with prednisolone/methylprednisolone was combined with additional immunosuppressant therapy. In 64.2% (27/42) of the non-responsive dogs, cobalamin supplementation was administered (Table 4). None of the dogs in the NRE outcome group, or any of the other treatment outcome groups, received treatment with albumin or blood products. 

## 4. Discussion

This large retrospective study showed that the period prevalence of CE was approximately 1% of the total number of dogs visiting two Swedish referral animal hospitals. The breeds with the highest RR of developing CE were Norwegian Lundehund, WHWT, and Miniature Poodle. An earlier age of CE onset was seen in French Bulldogs and Miniature Schnauzers, compared to the other breeds. As opposed to previous reports, most of the dogs were classified with IRE and the fewest with ARE. Dogs with NRE were more likely to present with hypoproteinemia, hypoalbuminemia, anemia, and ascites compared to the FRE, IRE, and non-classified outcome group. 

The period prevalence of CE was 1.1% in the present study, which corresponds to a previous study of 546 dogs performed at an animal hospital in the UK during a 5.5 year period (2003–2009), where the results indicated a period prevalence of 2% (546/27 463) for CE in dogs [6]. In another retrospective Italian study of 120 dogs, the period prevalence of dogs with signs consistent with CE was 0.9% (120/12 699) during 2013 [28]. 

The breeds identified with an increased RR of CE in this study were Norwegian Lundehund, WHWT, Miniature Poodle, Border Terrier, Rottweiler, Boxer, CKCS, French Bulldog, and Shetland Sheepdog. Norwegian Lundehunds are known to be predisposed to chronic gastrointestinal disease [13,17,29] and Rottweilers as well as Boxers have been reported to have an increased risk of developing CE in a former study from the UK [6]. Rottweilers have been described to be predisposed to eosinophilic enteritis [30,31], and Boxers and French Bulldogs have been reported to be susceptible to granulomatous colitis, caused by enteroinvasive *Escherichia coli* [8,32,33]. Boxers and WHWT were mentioned as two of the most commonly represented breeds in a retrospective study of 80 dogs with IBD in Scotland [20], and the results of a previous study from Switzerland indicated an increased proportion of WHWT with IRE and PLE [14]. To the authors’ knowledge, an increased RR of CE in the Miniature Poodle, Border Terrier, CKCS, and Shetland Sheepdog breeds have hitherto not been described. Even though German Shepherd dogs were one of the most represented breeds in the present study population, it was not one of the breeds that was found to have an increased RR of CE as reported in previous studies [6,7,10,11]. A possible reason that could partly explain this finding is that working dogs of the Swedish police force, mainly German Shepherd dogs, are routinely presented at these two animal hospitals for prophylactic interventions. This might lead to a potentially higher proportion of healthy German Shepherd dogs visiting these two animal hospitals than in other animal hospitals or referring clinics in Sweden, leading to a lower proportion of German Shepherd dogs with CE in the present study population.

In the present study, the overall median age of the dogs at the time of the first CE related visit was 3.8 years, which was lower than in other previous studies of 70 dogs from Switzerland and 80 dogs from Scotland: 5.3 years and 4.3 years, respectively [14,20]. There was no significant difference in age at presentation between the different classification groups in the present study. This differs from the results of the study from Switzerland, in which it was reported that dogs with FRE were significantly younger than dogs with IRE [14]. Another study of 203 dogs from the UK demonstrated that dogs in the ARE group were younger than dogs in the FRE and IRE group, respectively [25]. In the present study, the oldest dog in the FRE outcome group was twelve years old at the time of the first visit. This agrees with previous studies that have also reported that some dogs with FRE have presented at an old age: nine years and eleven years, respectively [3,34]. These results support the recommendation that a diet change should be considered regardless of the age of the dog. There was no significant difference in distribution between males and females in the present study population, which agrees with earlier studies (14, 20).

In the present study, the largest proportion of dogs was classified in the IRE outcome group (55.2%), which differs from earlier published studies. In previous studies, the FRE group typically has been the largest group with proportions larger than 50% [14,22,25]. In a retrospective study regarding canine CE in 203 dogs, 64% of the dogs were classified in the FRE group, 16.2% in the ARE group, and 19.2% were considered to have IRE [25]. One reason that could explain why the IRE outcome group is comparably large in the present study might be that both animal hospitals are referral hospitals and 16.2% of the dogs had been referred from general practitioners. It seems likely that many dogs that respond to a dietary intervention are not referred to an animal hospital for further investigation. 

The ARE outcome group contained the smallest number of dogs in the present study. In 43% (341/809) of the dogs, antibiotic treatment had been used at some time during their follow up period, but only 1% (8/814) of the dogs responded solely to antibiotic treatment and were therefore classified in the ARE outcome group. Among the dogs that received antibiotics, 46.2% (158/341) of the dogs were not assigned a classification due to difficulties to evaluate treatment response because of concurrent initial treatment with antibiotic and immunosuppressive therapy. The view of clinical use of antibiotic treatment may have shifted over time and become more restricted, which is reflected in changes in local standard operating procedures. The proportion of dogs with ARE in the present study is not in agreement with two earlier studies, where the ARE group constituted of 36.8% and 16.2% of the study populations respectively [3,25]. Swedish authorities also discourage long- term antibiotic treatment for dogs with CE, and immunosuppressive treatment is often started directly after the food trial if the response to diet change is inadequate and other causes of gastrointestinal disease have been excluded [35]. An important exception is the diagnosis of granulomatous colitis where enrofloxacin is recommended [35,36,37]. The use of different antibiotics has been described in dogs with CE, when food trial has failed, or if only a partial response to the diet change is observed [2,15,38,39,40]. The response, however, has shown to be short-lived after the antibiotic treatment has been terminated, with one study reporting relapse of the clinical signs within one month in 86% of the dogs treated with tylosin [41]. Some studies report remission of clinical signs with the use of metronidazole in dogs with CE, but the antibiotic treatment was combined with diet and other drugs which makes it difficult to interpret to what extent the response was due to the antibiotic treatment [15,23,40]. The relapse rate of CE signs after discontinuation of metronidazole has been reported to be 80% with the need for prolonged antibiotic treatment to control CE signs [2,3,25]. In one study, it was demonstrated that prednisone was as effective as a combined treatment with prednisone and metronidazole for induction therapy of canine IBD, putting the usefulness of metronidazole in treating CE in question [40]. The high relapse rate of dogs receiving antibiotics to treat CE can also be caused by an antibiotic-induced dysbiosis, as was reported for tylosin and metronidazole [42,43,44,45].

In the present study, 5.2% (42/814) of the dogs were classified in the NRE outcome group, and of these non-responsive dogs, 97.6% (41/42) were euthanized due to CE. In total, 14.3% (116/814) of dogs in the present study were euthanized due to CE. This result agrees with a retrospective study that reported that 13% (10/80) of the dogs were euthanized due to refractory IBD [20], even though some of the dogs had gone into remission before developing severe relapse and refractoriness to additional treatment. The result of the present study is also comparable to other previous studies where 19% (13/70) and 9% (15/165) of the dogs with CE had an intractable disease [14,26]. The long period of follow up in many dogs of this study enabled accurate assessments of the treatment response. Treatment response has been reported in several earlier studies, but the time of follow up is often less than three months [40,46,47,48,49,50,51,52], even though some studies have a longer follow up than six months [1,3,14,20,22,25,26,27]. 

In the present study, 17.4% of the dogs had hypoalbuminemia, and dogs with NRE were more likely to present with hypoalbuminemia compared to the FRE, IRE, and non-classified outcome groups (*p <* 0.008).) This corresponds to a previous study of 70 dogs with CE where hypoalbuminemia was shown to be associated with negative outcomes [14], as well as a previous study of 165 dogs with CE where non-survivors showed significantly lower serum albumin levels compared to survivors [26]. It is also comparable to the results of a previous study of 92 dogs with CE, that demonstrated that the proportion of dogs with normal serum albumin levels was significantly higher in the IRE group than in the NRE group [27]. However, no significant differences in proportion of dogs with hypoalbuminemia were found between the FRE and the IRE outcome group in the present study, which was demonstrated in a previous study of 203 dogs with CE [25]. A possible explanation for this finding is that the CE of the dogs with IRE in the present group was not as severe as the CE of the IRE group in the previous study.

Dogs in the NRE outcome group in the present study were more likely to present with anemia compared to the dogs with FRE, IRE, and the non-classified outcome group, which has also been described in a previous study [53]. Non-responsive dogs also presented with an increased CRP compared to the IRE outcome group, but not compared to the FRE- or non-classified outcome group. Increased CRP has been described as a marker of disease severity in dogs with CE in a previous study [18], but was not significantly associated with negative outcomes in another previous study [14]. Hypocobalaminemia was reported in 15.1% (98/647) of the dogs in the present study. Dogs with NRE were not more likely to present with hypocobalaminemia, as opposed to an earlier study where hypocobalaminemia was associated with negative outcomes [14]. In the present study, a higher proportion of dogs received supplementation with cobalamin compared to the proportion that presented with low cobalamin levels. Substituting dogs with serum cobalamin concentrations close to the lower reference range originates from studies revealing that affected dogs can have subtle cobalamin deficiency as manifested by increased serum methylmalonic acid concentrations despite cobalamin concentrations within the reference range, and that they respond with an increase in serum cobalamin after oral vitamin substitution [54,55].

Because of the retrospective nature of the study, the information in the medical records was sometimes incomplete. Hematology and biochemistry results were not accessible in all dogs, and it was not possible to assess retrospectively the two clinical indexes for disease activity that have been defined previously: CIBDAI and CCECAI [14,18]. Another limitation is that treatment response was subjectively evaluated from the information that was documented in the medical record by the attending clinician. 

## 5. Conclusions

In conclusion, the period prevalence of dogs with CE at two Swedish referral animal hospitals was 1.1%, which is comparable to results of previous studies. Some breeds that were identified with an increased RR of CE in the present study have previously been described as risk breeds, but Miniature Poodle, Border Terrier, CKCS, and Shetland Sheepdog have, to the authors’ knowledge, not previously been described as breeds with an increased risk of CE. Compared to the other breeds, onset of CE was identified at an earlier age in French Bulldogs and Miniature Schnauzers. Hydrolyzed diet and immunosuppressive treatment consisting of prednisolone/methylprednisolone were the most common treatment alternatives. Most dogs with CE were categorized in the IRE outcome group and the smallest proportion of dogs in the ARE outcome group. Even though antibiotic treatment had been used in a comparably large proportion dogs, only 1.1% of the dogs were classified in the ARE outcome group, which differs from previous studies. Dogs with NRE were more likely to present with hypoproteinemia, hypoalbuminemia, anemia, and ascites compared to the dogs with FRE, IRE, and the non-classified outcome group.

## Figures and Tables

**Table 1:** Summary of signalment variables, presenting signs, and follow up information by treatment outcome group.

	Information Rate (*n* = 814)	Variable	Overall (*n* = 814)	Food Responsive (*n* = 93)	Immunosuppressant Responsive (*n* = 449)	Antibiotic Responsive (*n* = 9)	Non Responsive (*n* = 42)	Non Classified (*n* = 221)
Study population	814/814	Sex (male/female)	463/351	52/41	246/203	6/3	23/19	136/85
	113/814	Median BCS (1- 9/9)	4 (3–5)	4 (4–5)	4 (4–5)	4(4)	3 (3–4)	4 (3–4)
	702//814	Median body weight (kg)	11.1 (6.5–25.9)	12.3 (6.4–27.6)	10.0 (6.3–23.7)	9.0 (6.2–14.1)	12.2 (7.6–30.2)	15.3 (7.5–28.9)
	814/814	Median age at first visit (years)	3.8 (1.8–6.7)	3.8 (1.5–6.0)	3.7 (1.8–6.6)	4.1 (0.8–8.1)	5.0 (3.5–9.1)	3.9 (1.9–6.8)
	814/814	Median age at last visit (years)	7.6 (4.8–10)	7.5 (4.8–9.3)	7.9 (5.5–10.6)	8.0 (2.5–12.8)	6.4 (3.7–9.1)	7.0 (4.2–9.5)
	251/814	Median age at death (years)	9.2 (6.5–11.5)	8.9 (6.9–10.8)	10.3 (7.5–12.4)	11.3 (7.9–14.8)	6.6 (3.8–9.2)	8.8 (6.6–11.2)
	814/814	Median time of follow up (years)	2.8 (0.9–4.6)	3.1 (1.1–4.5)	3.3 (1.5–5.2)	3.3 (1.7–4.3)	0.3 (0.1–0.7)	2.1 (0.4–3.6)
	244/814	Euthanasia because of CE (yes/no)	116/128	3/9	42/82	1/1	41/1	29/35
Presenting signs	759/814	Vomiting (yes/no)	598/161	64/25	350/71	5/4	29/8	150/53
	764/814	Diarrhea (yes/no)	574/190	67/25	298/115	8/1	35/5	166/44
	503/814	Anorexia (yes/no)	282/221	33/32	157/108	2/6	21/10	69/65
	269/814	Weight loss (yes/no)	184/85	14/14	93/47	1/1	22/2	54/21
	469/814	Activity (decreased/normal)	247/222	29/29	124/114	1/5	21/13	72/61
	764/814	Hematochezia (yes/no)	31/733	5/87	16/398	0/9	2/38	8/201
	67/814	Stool frequency (increased/normal)	41/26	8/6	17/10	2/0	3/1	11/9
	250/814	Pruritus (yes/no)	205/45	17/12	122/17	2/0	6/3	58/13

**Table 2:** Summary of laboratory results and ultrasonographic findings in the gastrointestinal (GI) tract by treatment outcome group.

	Information Rate (*n* = 814)	Variable	Overall (*n* = 814)	Food Responsive (*n* = 93)	Immunosuppressant Responsive (*n* = 449)	Antibiotic Responsive (*n* = 9)	Non Responsive (*n* = 42)	Non Classified (*n* = 221)
Blood samples	814/814	Blood samples taken (yes/no)	792/22	91/2	438/11	9/0	42/0	212/9
Blood sample results	814/814	Blood sample results available (yes/no)	774/40	90/3	428/21	8/1	42/0	206/15
	632/814	Anemia (yes/no)	32/600	2/70	14/333	1/6	8/31	7/160
	637/814	Leukocytosis (yes/no)	65/572	5/66	31/320	1/6	11/28	17/152
	637/814	Leukopenia (yes/no)	18/619	0/71	11/340	0/7	0/39	7/162
	218/814	Eosinophilia (yes/no)	32/186	6/23	14/85	0/3	2/20	10/55
	267/814	Elevated CRP (yes/no)	145/122	26/19	52/72	1/0	20/2	46/29
	608/814	Elevated creatinine (yes/no)	13/595	1/70	5/331	0/7	3/31	4/156
	486/814	Elevated urea (yes/no)	28/458	3/44	15/276	0/4	0/19	10/115
	621/814	Elevated ALT (yes/no)	105/516	10/64	67/277	0/7	6/29	22/139
	126/814	Hypocalcemia (total) (yes/no)	15/111	1/19	10/53	0/1	2/3	2/35
	647/814	Hypocobalaminemia (yes/no)	98/549	6/75	53/294	0/4	7/29	32/147
	638/814	Low folate (yes/no)	194/444	18/62	122/221	0/4	6/29	48/128
	655/814	Hypoproteinemia (yes/no)	125/530	13/66	61/292	2/5	19/19	30/148
	662/814	Hypoalbuminemia (yes/no)	116/546	7/73	59/294	0/7	20/21	30/151
Other clinical signs	550/814	Ascites (yes/no)	37/513	1/67	20/279	0/5	10/24	6/138
Diagnostic imaging	814/814	Abdominal ultrasound (yes/no)	563/251	69/24	305/144	5/4	36/6	148/73
	563/814	Ultrasonographic abnormalities GI tract (yes/no)	335/228	38/31	199/106	3/2	23/12	72/77

**Table 3:** Summary of gastrointestinal (GI) endoscopy findings and histological findings by treatment outcome group.

	Information Rate (*n* = 814)	Variable	Overall (*n* = 814)	Food Responsive (*n* = 93)	Immunosuppressant Responsive (*n* = 449)	Antibiotic Responsive (*n* = 9)	Non Responsive (*n* = 42)	Non Classified (*n* = 221)
GI endoscopy	814/814	GI endoscopy (yes/no)	606/208	34/59	379/70	8/1	24/18	161/60
	594/814	Macroscopic abnormalities (yes/no)	593/1	30/1	371/0	8/0	24/0	160/0
	594/814	Histological abnormalities (yes/no)	576/18	28/3	362/9	8/0	23/1	155/5
Histological results stomach	566/814	Normal stomach (yes/no)	82/484	8/22	36/318	0/7	4/17	34/120
	566/814	Chronic gastritis (yes/no)	337/229	15/15	224/130	4/3	10/11	84/70
	566/814	Lymphoplasmacytic gastritis (yes/no)	48/518	1/29	34/320	2/5	3/18	8/146
	566/814	Eosinophilic gastritis (yes/no)	95/471	6/24	59/295	1/6	2/19	27/127
	566/814	Neoplasia (yes/no)	4/562	0/30	1/353	0/7	2/19	1/153
Histological results duodenum	493/814	Normal duodenum (yes/no)	106/387	6/17	63/253	4/2	1/17	32/98
	493/814	Chronic enteritis (yes/no)	168/325	9/14	108/208	2/4	5/13	44/86
	493/814	Lymphoplasmacytic enteritis (yes/no)	95/398	3/20	58/258	0/6	7/11	27/103
	493/814	Eosinophilic enteritis (yes/no)	123/370	5/18	86/230	0/6	5/13	27/103
	493/814	Neoplasia (yes/no)	1/492	0/23	1/315	0/6	0/18	0/130
Histological results colon	356/814	Normal colon (yes/no)	32/324	4/12	18/197	0/6	2/14	8/95
	356/814	Chronic colitis (yes/no)	204/152	8/8	130/85	3/3	8/8	55/48
	356/814	Lymphoplasmacytic colitis (yes/no)	53/303	2/14	27/188	0/6	4/12	20/83
	356/814	Eosinophilic colitis (yes/no)	65/291	2/14	40/175	1/5	2/14	20/83
	356/814	Granulomatous colitis (yes/no)	2/354	0/16	0/215	2/4	0/16	0/103
	356/814	Neoplasia (yes/no)	0/356	0/16	0/215	0/6	0/16	0/103

**Table 4:** Summary of treatments used for the dogs in the study population as well as information concerning co-morbidities by treatment outcome group. Other immunosuppressive treatment include cyclosporine, azathioprine, chlorambucil, budesonide, and sulfasalazine/olsalazine. Gastroprotectant treatment included proton pump inhibitors, sucralfate, and/or famotidine. Notice that a dog may have received more than one of the listed medical treatments.

	Variable	Overall (*n* = 814)	Food Responsive (*n* = 93)	Immunosuppressant Responsive (*n* = 449)	Antibiotic Responsive (*n* = 9)	Non Responsive (*n* = 42)	Non Classified (*n* = 221)
Treatment	Hydrolyzed diet	563/814	49/93	323/449	7/9	26/42	158/221
	Novel protein diet	106/814	19/93	57/449	1/9	5/42	24/221
	Gastrointestinal diet	111/814	23/93	50/449	1/9	8/42	29/221
	Home cooked diet	18/814	0/93	10/449	0/9	1/42	7/221
	Other diet	14/814	2/93	9/449	0/9	2/42	1/221
	Prednisolone/methylprednisolone	668/814	6/93	446/449	7/9	39/42	170/221
	Budesonide	44/814	0/93	23/449	0/9	2/42	19/221
	Cyclosporine	81/814	1/93	50/449	0/9	8/42	22/221
	Chlorambucil	11/814	0/93	2/449	0/9	2/42	7/221
	Sulfasalazine/olsalazine	20/814	0/93	4/449	2/9	4/42	10/221
	Prednisolone/methylprednisolone combined with other immunosuppressive treatment	132/814	1/93	72/449	2/9	15/42	42/221
	Antibiotic treatment	341/814	8/93	138/449	9/9	28/42	158/221
	Gastroprotectant treatment	512/814	48/93	299/449	5/9	28/42	132/221
	Cobalamin supplementation	380/814	36/93	216/449	3/9	27/42	98/221
	Folic acid supplementation	189/814	21/93	111/449	1/9	12/42	44/221
	Probiotics	91/814	5/93	46/449	2/9	7/42	31/221
Other diseases/medication	Other diseases	453/814	56/93	266/449	5/9	15/42	111/221
	Other medication	281/814	18/93	158/449	4/9	20/42	81/221

## Data Availability

The data presented in this study are available on request from the corresponding author.
